# Preconditioning with Lipopolysaccharide or Lipoteichoic Acid Protects against *Staphylococcus aureus* Mammary Infection in Mice

**DOI:** 10.3389/fimmu.2017.00833

**Published:** 2017-07-24

**Authors:** Koen Breyne, Jonas Steenbrugge, Kristel Demeyere, Tom Vanden Berghe, Evelyne Meyer

**Affiliations:** ^1^Biochemistry, Faculty of Veterinary Medicine, Department of Pharmacology, Toxicology and Biochemistry, Ghent University, Merelbeke, Belgium; ^2^Peter Vandenabeele Lab, Inflammation Research Center, Department of Biomedical Molecular Biology, VIB, Ghent University, Zwijnaarde, Belgium

**Keywords:** intramammary injection, *Staphylococcus aureus*, mastitis, lipopolysaccharide, lipoteichoic acid, chitinase 3-like 1, lipocalin 2

## Abstract

*Staphylococcus aureus* is one of the most causative agents of mastitis and is associated with chronic udder infections. The persistency of the pathogen is believed to be the result of an insufficient triggering of local inflammatory signaling. In this study, the preclinical mastitis model was used, aiming to evaluate if lipopolysaccharide (LPS) or lipoteichoic acid (LTA) preconditioning could aid the host in more effectively clearing or at least limiting a subsequent *S. aureus* infection. A prototypic Gram-negative virulence factor, i.e., LPS and Gram-positive virulence factor, i.e., LTA were screened whether they were able to boost the local immune compartment. Compared to *S. aureus*-induced inflammation, both toxins had a remarkable high potency to efficiently induce two novel selected innate immunity biomarkers i.e., lipocalin 2 (LCN2) and chitinase 3-like 1 (CHI3L1). When combining mammary inoculation of LPS or LTA prior to a local *S. aureus* infection, we were able to modulate the innate immune response, reduce local bacterial loads, and induce either LCN2 or CHI3L1 at 24 h post-infection. Clodronate depletion of mammary macrophages also identified that macrophages contribute only to a limited extend to the LPS/LTA-induced immunomodulation upon *S. aureus* infection. Based on histological neutrophil influx evaluation, concomitant local cytokine profiles and LCN2/CHI3L1 patterns, the macrophage-independent signaling plays a major role in the LPS- or LTA-pretreated *S. aureus*-infected mouse mammary gland. Our results highlight the importance of a vigilant microenvironment during the innate immune response of the mammary gland and offer novel insights for new approaches concerning effective immunomodulation against a local bacterial infection.

## Introduction

Antibiotic-resistant bacteria remain a serious problem in modern health care, food safety, and animal health, resulting in an urgent need for alternatives to replace the conventional curative treatment (1, 2). The ideal strategy would be that (mis)used antibiotics become obsolete or can at least be partly replaced by preventive treatment. The latter strategy modulates the immune system by a primary insult that enhances the activity of a beneficial antibacterial host response which, in turn, clears the bacterial hazard when it presents itself (3). In contrast to previous literature, it is now more accepted that next to the adaptive immunity, the innate immunity can also harbor some immunological memory (4).

Mastitis is a disease featured by either chronically or acutely inflamed mammary glands and is still a major challenge in the dairy sector as it affects both milk quality and animal welfare (5, 6). *Staphylococcus aureus* (*S. aureus*) in the udder of dairy cows causes harmful clinical symptoms and is associated with poor cure rates (7). The insufficient recognition of the infecting *S. aureus* in the mammary compartment is believed to hinder the host in raising an adequate innate immune response and the pathogen may, therefore, persist in the tissue (8, 9). In addition, these Gram-positive bacteria may infiltrate the mammary epithelial cell (MEC) layers and induce complicating factors such as biofilms formation (10, 11). Altogether, these observations are recognized to be a reason why classical antibiotic therapies have difficulties in eradicating contagious infections from a herd. Moreover, this currently leads to excessive use of antibiotics and even drug-resistant variants of mastitis pathogens (12–14). Commercial efforts have been made to create *S. aureus* mastitis vaccines e.g., Lysigin (Boehringer Ingelheim Vetmedica, Inc.) and Startvac (Hipra, Inc.) as an alternative to antibiotics. Unfortunately, many of these strategies have shown limited efficacy under field conditions (15).

The mammary gland is an immune receptive organ that harbors different kinds of pathogen recognition receptors (PRRs) on local cells (16). It is accepted that the outcome of the inflammatory reaction in this niche strongly depends on the efficiency of activating local PRR-expressing cells through the recognition of pathogen-associated molecular patterns (PAMPs) (17). Gram-negative mastitis pathogens contain several highly potent PAMPs that trigger multiple PRRs during an intramammary infection and result in a cytokine storm associated with acute symptoms at the cow’s udder. These events eventually induce either a fast recovery or a negative outcome (18, 19). By contrast, the comprehensive identification of Gram-positive pathogens by the innate immune system is impeded due to a smaller number of highly potent immunodominant proteins that typically mediate much milder symptoms and chronic infections (20–23). Unraveling the inflammatory mechanisms that are triggered by different bovine mastitis pathogens and boost the host’s immunity for low immunopotent bacterial strains such as *S. aureus* remain, therefore, mandatory. Such experiments in cows are severely limited with respect to availability of immunological research tools and requested infrastructure. Complementing *in vitro* bovine models, *in vivo* mouse models were developed to tackle these major drawbacks (24). The latter preclinical models allow the dynamic interaction between mammary gland cell populations and have been successfully used with different mastitic bacteria, including bovine *S. aureus* isolates, focusing on the early innate immune response that is highly conserved between both mammalian species (25).

Until recently, the main focus with regard to PRR-containing mammary cells was on the MECs as these were expected to be the key cell type responsible for sensing of mastitis pathogens (17, 26). Nevertheless, novel data indicate that the mammary compartment is an immunological microenvironment where multiple innate immune cells are present that are also able to sense virulence factors of pathogens (27). The differential role of the sentinel cell types responsible for immunosurveillance during an infection is gaining increasing importance in the mammary gland immunity field (28–32). It is also currently, albeit partly, known that the mammary epithelium communicates with other PRR-containing cells in the mammary gland for bacterial recognition, e.g., by shedding PAMPs such as CD14 and likely also through microRNA (33, 34). However, whether appropriate activation of this microenvironment may trigger subsequent antibacterial or anti-inflammatory responses to a secondary insult remains largely unknown.

The aim of this paper was to analyze the antibacterial capacity of the mammary microenvironment against *S. aureus* mastitis using an intraductal inoculation mouse model. First, the innate immune response of the mammary compartment resulting from exposure to virulence factors lipopolysaccharide (LPS) and lipoteichoic acid (LTA) or the well characterized bovine mastitis isolate *S. aureus* Newbould 305 was investigated. Second, we assessed immunomodulation and the antibacterial response of the complete mammary microenvironment by LPS or LTA on a subsequent insult with *S. aureus*. Third, we verified whether the preconditioning by either LPS or LTA to a *S. aureus* infection in our mouse model was unique for the local macrophages or whether other MECs are of essence for this beneficial response.

For the analysis of the host immune response in the different experiments, we preferred to measure lipocalin 2 (LCN2) and chitinase 3-like 1 (CHI3L1) to complement local cytokine profiles, rather than classical acute phase protein, such as serum amyloid A and haptoglobin. Both LCN2 and CHI3L1 are increasingly reported as key players in a wide range of inflammatory diseases. LCN2 binds and sequesters the iron-scavenging siderophore enterobactin, preventing Gram-negative bacterial iron acquisition (35, 36). Recently, it has been shown that secreted human LCN2 cloned into bovine mammary epithelial stem cells actively inhibits growth of mastitis-causing *S. aureus* as well (37). CHI3L1, on the other hand, belongs to the family of chitinase-like proteins and was initially reported in host antifungal responses as an important scavenger of chitin that is present in the fungal cell wall (38). However, further research demonstrated that the enzymatically inactive CHI3L1 has prominent functions in bacterial adhesion and invasion in host tissue, bacterial clearance, and innate immunity processes (39–43).

## Materials and Methods

### Intramammary Inoculation Model

**Figure 1:**
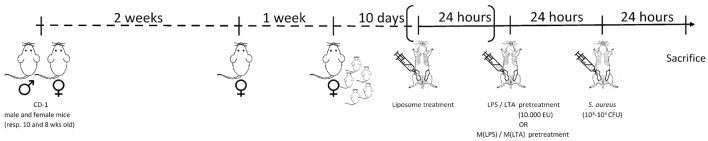
Experimental design. Hsd:ICR (CD1) 8-week-old female outbred mice were mated with 10-week-old male mice. The female mice were isolated for 1 week to give birth to their offspring. To ease the intraductal accessibility of the lactating mothers, the 10-day-old pups were weaned 2 h before the first inoculation. Depending on the preconditioning experiment: lipopolysaccharide (LPS)/lipoteichoic acid (LTA)/PBS or *ex vivo* LPS-/LTA-/PBS-treated macrophages were injected 24 h prior to the induction of *Staphylococcus aureus* mastitis in mice. To deplete local alveolar macrophages, preconditioning experiments could be preceded by clodronate liposome treatment. All intraductal injections (100 µl) were performed in the fourth gland pair of each of the mice. At relevant time points, post-inoculation mice were sedated and subsequently euthanized through cervical dislocation. Mammary glands were harvested to determine local bacterial loads, cytokine levels, or histopathology.

Hsd:ICR (CD1) outbred mice were purchased from a commercial supplier (ENVIGO, The Netherlands). The mice were fed *ad libitum* in a controlled environment that included light and dark cycles (12 h light: 12 h darkness). All the protocols that involved animals were approved by the Committee on the Ethics of Animal Experiments of the Faculty of Veterinary Medicine, Ghent University (permit number 2014/107 and 2015/127). The experimental design of this study is shown in Figure 1. Eight-week-old female mice were mated with 10-week-old male mice. To ease the intraductal accessibility of the lactating mothers, the 10-day-old pups were weaned 2 h before the first inoculation. If possible the pups were transferred to foster mothers, the remaining pups were culled by CO_2_ in a closed compartment as approved by the ethical committee of the faculty. All intraductal injections (100 µl) using a 32-gauge blunt needle were performed in the fourth gland pair of each of the mice under isoflurane anesthesia combined with the long-acting analgesic buprenorphine (10 µg/kg Vetergesic, Patheon UK Ltd., UK) and core body temperature was post-operative monitored with a rectal thermistor. Next to the ones described below, different agents were injected in the mammary gland, such as clodronate and PBS liposomes (Van Rooijen, The Netherlands). Inoculation of mammary glands with clodronate-containing liposomes depletes local alveolar macrophages and indirectly also decreases the recruitment of PMNs following intramammary LPS inoculation (27). At relevant time points post-inoculation (p.i.) mice were sedated by an intraperitoneal administered mixture of ketamine (100 mg/kg Anesketin, Eurovet Animal Health BV, Bladel, The Netherlands) and xylazine (10 mg/kg; Xylazini Hydrochloridum, Val d’Hony-Verdifarm, Belgium) and, subsequently, euthanized through cervical dislocation. Mammary glands were harvested to determine local bacterial loads, cytokine levels, or histopathology.

### Bacterial Products

The mastitis isolate *S. aureus* Newbould 305 (ATCC 29740) was grown in brain heart infusion (Thermo Scientific, Belgium) broth for 5 h from a frozen stock. The CFU/mL was determined after 24 h of incubation at 37°C from the 1/100 diluted bacterial preculture by spectrophotometric measurements at 600 nm (OD600) and were confirmed *a posteriori* by plating on tryptic soy agar (Thermo Scientific, Belgium) plates. The pathogen was suspended in PBS to achieve the required concentration (10^3^–10^4^ CFU/100μl). This 100 µl solution was then used to inoculate the mammary gland in the different mouse experiments (Figure 1). Ultrapure LPS 0111:B4 (InvivoGen, USA) and ultrapure LTA from *S. aureus* (InvivoGen, USA) were suspended in endotoxin-free water and diluted to the required concentration in PBS. These 100 µl LPS and LTA solutions were injected 24 h prior to the intramammary infection with *S. aureus* (Figure 1).

### Cell Culture

Peritoneal macrophages were isolated as described in Walachowski et al. (44) and plated out in DMEM/F12 (Gibco) with 10% fetal calf serum for 2 h according to Mosser and Edwards (45). The non-attached cells are washed out and the sticky macrophages were trypsinized, washed, and checked for their F4/80 (clone CI:A3-1AbD Serotec) positivity on a flow cytometer (Cytoflex, Beckman Coulter). Subsequently, macrophages were activated by exposing them *in vitro* to 10,000 EU LPS [referred to as M(LPS)], 10,000 EU LTA [referred to as M(LTA)], or PBS [referred to as M(PBS)] for 24 h (45). Cells were then washed twice with PBS. As shown in the experimental design flow (Figure 1), these treated macrophages were injected in the mammary gland 24 h prior to a mammary infection with *S. aureus*.

### Quantitative Cytokine Array, LCN2, and CHI3L1 ELISA

Hundred microliters of mammary gland homogenate were mixed with 300 µl lysis buffer supplemented with protease inhibitors (200 mM NaCl, 10 mM Tris–HCl pH 7.4, 5 mM EDTA, 1% Nonidet P-40, 10% glycerol, 1 mM oxidized l-glutathione, 100 µM PMSF, 2.1 µM leupeptin, and 0.15 µM aprotinin) to extract cellular proteins. The suspensions rested overnight at −20°C, were centrifuged at 12,250 × *g* for 1 h, and finally the supernatant was centrifuged for another 30 min to obtain a clear pellet. Protein concentration of the lysate samples was determined as previously described (46). Cytokine quantification (ProcartaPlex from eBioscience), LCN2 (Biotechne, USA), and CHI3L1 ELISA (Biotechne, USA) on the mammary gland lysates were all performed according to company guidelines or previously reported (47).

### *In Vivo* Imaging of Activated PMN

When PMN are activated in inflamed tissues, myeloperoxidase (MPO) gets released and catalyzes the reaction of H_2_O_2_ and chloride ions (Cl^−^) to produce hypochlorous acid (HOCI) (48). In turn, HOCI can oxidize luminol (5-amino-2,3-dihydrophthalazine-1,4-dione), which triggers chemiluminescence. MPO can also directly utilize superoxide anion as a substrate for peroxidase-catalyzed oxidation of luminol (45). To visualize PMN activation in our model, we injected 10 mg/ml luminol (Sigma-Aldrich, USA) i.p. and detected the bioluminescent signal with the IVIS lumina II (Caliper) (49).

### Histopathology

Two mammary glands per condition were fixed in buffered 3.5% formaldehyde for 24 h at room temperature, dehydrated, and embedded in paraffin wax. Sections were deparaffinized, hydrated, and stained with hematoxylin and eosin. Subsequently, sections were dehydrated and mounted with a cover glass.

### Statistical Analysis

Statistical differences were determined on normalized data with an independent sample *T*-test (SPSS 20.0, IBM Corporation, USA). The statistical differences for multiple comparisons were determined using ANOVA tests. Statistical significance between different groups was determined through Tukey *post hoc* testing.

## Results

### Both LPS, and to a Lesser Extent Also LTA, Induce Mammary Immune Cell Recruitment and Local IL-8 Induction

**Figure 2:**
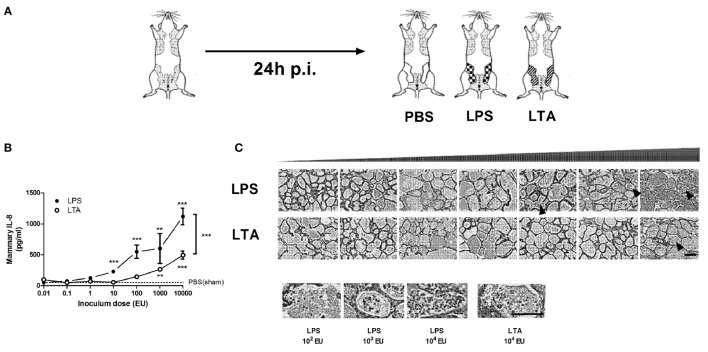
Effect of an intramammary injection with lipopolysaccharide (LPS) and lipoteichoic acid (LTA) on neutrophil recruitment in the intraductal mouse model. **(A)** Schematic presentation of the set-up. The white mammary glands represent the PBS (sham)-inoculated mammary glands, whereas the patterned glands represent the glands inoculated with LPS or LTA. **(B)** IL-8, a prototype chemoattractants for PMN influx was determined in mammary gland tissue 24 h p.i. of different doses of LPS and LTA (for inoculum dose 0.01, 0.1, 1, 10, and 10,000 EU LPS/LTA: *n* = 3 mice/6 glands; for inoculum dose 100 and 1,000 EU LPS/LTA: *n* = 2 mice/4 glands). Represented data were pooled from two independent experiments. Values are expressed as mean ± SEM. Significance between LPS-treated glands and PBS (sham)-inoculated mammary glands or significance between LTA-treated glands and PBS (sham)-inoculated mammary glands is illustrated without brackets. Values are expressed as mean ± SEM. **P < 0.01 and ***P < 0.001 versus sham. Significance between LPS- and LTA-inoculated glands is illustrated with brackets. Values are expressed as mean ± SEM. ***P < 0.001. **(C)** Mammary tissue was evaluated histologically p.i. with different doses of LPS or LTA. Immune cells in the alveolar lumen are indicated with black arrows and enlarged in the images below. Scale bar, 100 µm. p.i., post-inoculation.

The effect of intramammary challenge with different doses (0.01–10,000 EU) of either LPS or LTA was compared in mice at 24 h p.i. (Figure 2A). Mammary glands subjected to LPS at 10,000 EU had significantly higher IL-8 levels than glands exposed to a same dose of LTA 24 h p.i. (Figure 2B). While significant differences were detected in local IL-8 levels after a low-dose challenge of LPS (10 EU) compared to PBS (sham)-inoculated glands, IL-8 levels only increased in LTA challenged glands starting at a 100-fold higher dose (1,000 EU). This observation was in line with the histological data, showing the recruitment of immune cells in the alveolar lumen at a later time point for the mammary glands exposed to LTA compared to the mammary glands injected with LPS (Figure 2C).

### LPS Treatment More Effectively Induces Local LCN2 and CHI3L1 than LTA Treatment, Partially Concomitant with Mammary Immune Cell Recruitment

**Figure 3:**
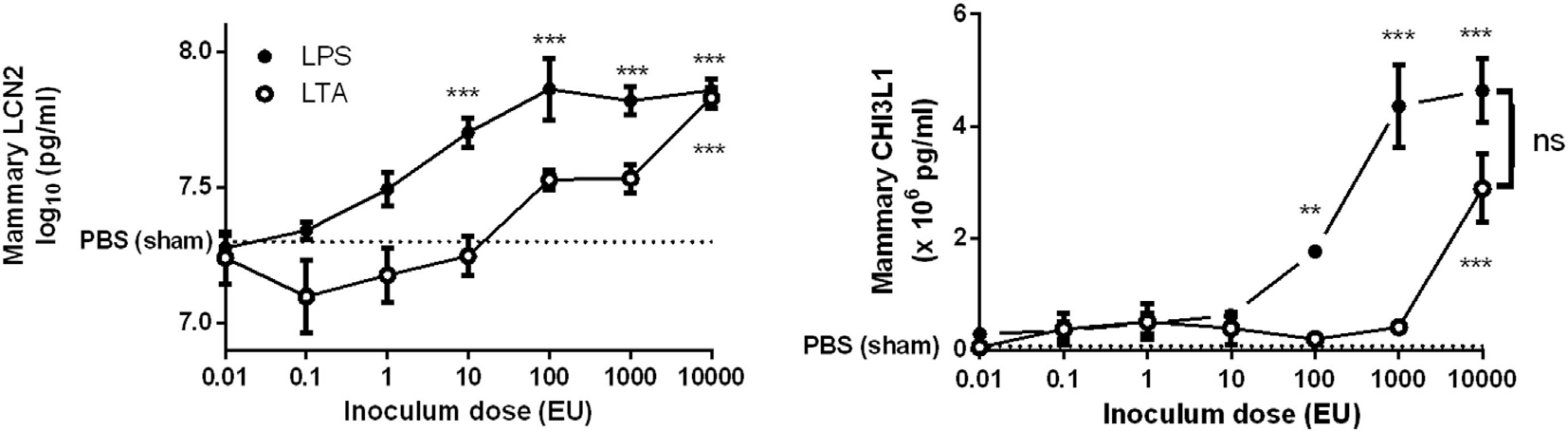
Effect of an intramammary injection with lipopolysaccharide (LPS) and lipoteichoic acid (LTA) on local lipocalin 2 (LCN2) and chitinase 3-like 1 (CHI3L1) levels in the intraductal mouse model. LCN2 and CHI3L1 levels were determined in mammary gland tissue 24 h p.i. of different doses of LPS and LTA (*n* = 3 mice/6 glands for inoculum dose 0.01, 0.1, 1, 10, and 10,000 EU LPS/LTA; *n* = 2 mice/4 glands for inoculum dose 100 and 1,000 EU LPS/LTA). Represented data were pooled from two independent experiments. p.i., post-inoculation. Values are expressed as mean ± SEM (**P < 0.01 and ***P < 0.001 versus sham).

Lipopolysaccharide was more effective than LTA to significantly induce both mammary LCN2 and CHI3L1 compared to PBS (sham)-inoculated mammary glands (Figure 3). Nevertheless, it was also observed that local LCN2 and CHI3L1 levels not fully mimicked the previously mentioned IL-8 levels. Indeed, dissimilar to the IL-8 patterns, 10-fold higher doses of both LPS (≥100 EU) and LTA (10,000 EU) were needed to significantly induce CHI3L1 in the mammary gland compared to sham-inoculated glands. Furthermore, no significant difference in LCN2 and CHI3L1 levels could be observed between the LPS and LTA inductions at the highest dose (10,000 EU). Interestingly, recruitment of neutrophils was observed in the mammary alveoli at the time point that both the LCN2 and CHI3L1 levels in the LTA and LPS groups were significantly increased compared to the PBS (sham)-inoculated glands.

### *S. aureus* Effectively Recruits Immune Cells during Mammary-Induced Inflammation Independently of LCN2

**Figure 4:**
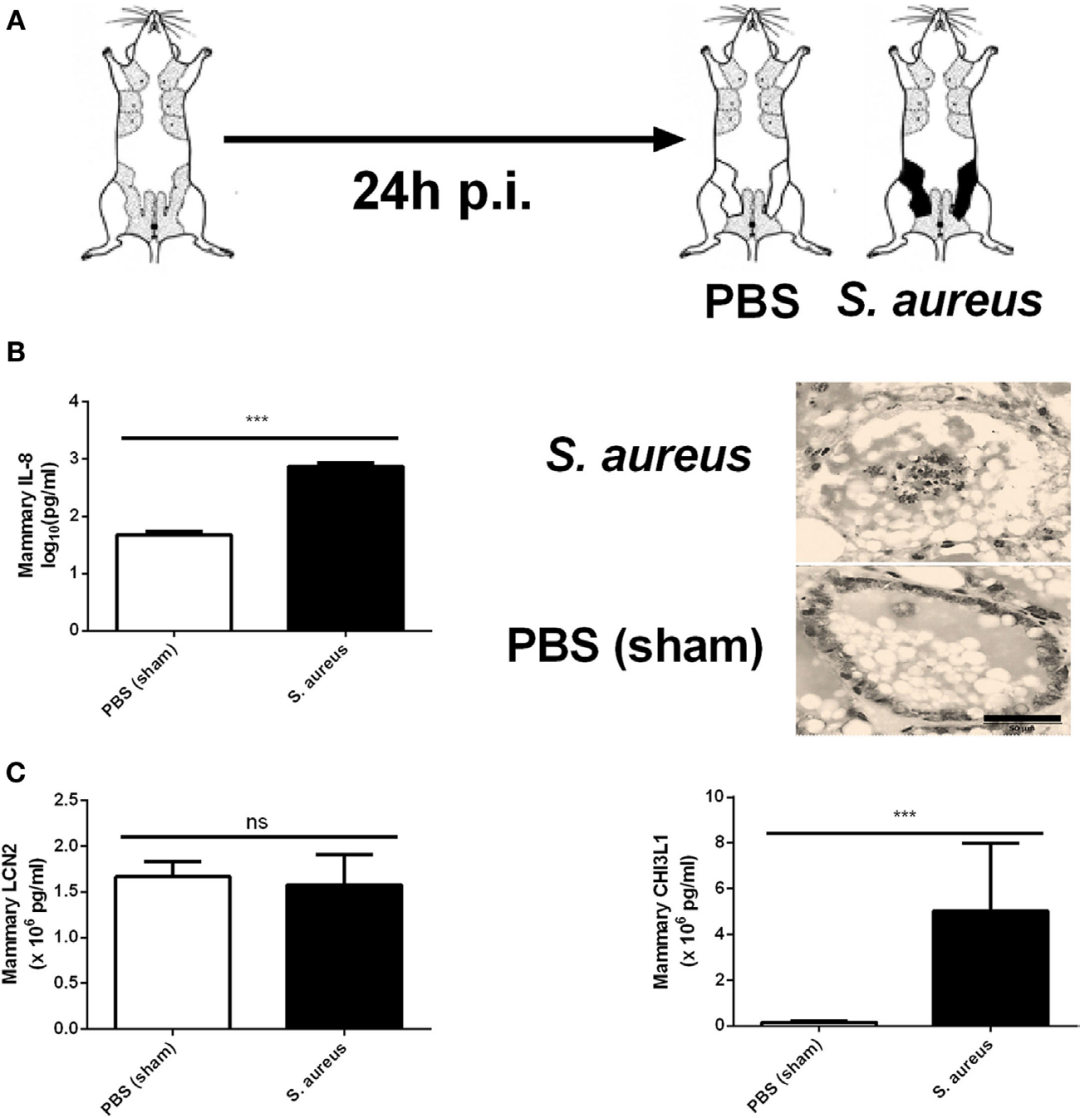
Inflammatory response to an intramammary infection with *Staphylococcus aureus* in mice. **(A)** Schematic presentation of the experimental set-up. The white mammary glands represent the PBS (sham)-inoculated mammary glands, whereas the black glands represent the glands inoculated with *S. aureus*. **(B)** Quantitative levels of IL-8 determined in mammary gland tissue to PBS (sham) and *S. aureus* 24 h p.i. (left) (PBS inoculation: *n* = 10 glands; *S. aureus* inoculation: *n* = 8 glands). Representative images of alveoli of mammary glands exposed to PBS (sham) (bottom) and *S. aureus* (top) 24 h p.i. (right). Scale bar, 20 µm. **(C)** Lipocalin 2 and chitinase 3-like 1 levels were determined in mammary gland tissue 24 h after intramammary exposure with PBS or *S. aureus* (PBS inoculation: *n* = 6 glands; *S. aureus* inoculation: *n* = 7 glands). Represented data were pooled from three independent experiments. p.i., post-inoculation. Values are expressed as mean ± SEM (***P < 0.001).

In contrast to sterile mammary gland-induced inflammation, inoculation of 1,000 CFU *S. aureus* Newbould 305 (*S. aureus*) in the murine mammary gland resulted in high bacterial counts (9.2 ± 0.3 log_10_ CFU/g mammary gland), accompanied by a prominent local host response at 24 h p.i. (Figure 4A). This inflammatory response was characterized by the production of multiple cytokines and chemokines as comparing tissue samples of a *S. aureus*- and a PBS (sham)-inoculated murine mammary gland (data not shown). In addition, the significant increase of IL-8 levels at 24 p.i. in the *S. aureus*-infected glands compared to sham-inoculated control glands could be demonstrated (Figure 4B, left). This latter observation could be confirmed upon histological examination through a pronounced presence of immune cells in the lumen of the mammary alveoli with *S. aureus*, compared to sham-inoculated mammary glands (Figure 4B, right). Interestingly, the IL-8 levels showed a median fold increase of 14.0 between *S. aureus*- and sham-inoculated mammary glands, whereas a 34.1 and 14.6 median fold increase was observed for 10,000 EU LPS- or LTA-exposed mammary glands, respectively, compared to the control glands. Although LCN2 levels in these infected glands were not significantly different from the LCN2 levels in sham-inoculated control glands, local induction of CHI3L1 levels by *S. aureus* could still be observed at 24 h p.i. (Figure 4C). More specifically, the median fold increase of local CHI3L1 levels in *S. aureus*-inoculated glands compared to sham-inoculated control glands was 2.88, which is an order of magnitude lower compared to the CHI3L1 levels following exposure to 10,000 EU LPS or LTA (8.16 and 5.13, respectively).

### Preconditioning of the Complete Mammary Gland Compartment with LPS or LTA Strongly Immunomodulates *S. aureus* Mastitis, Reducing Bacterial Loads, Recruiting PMN, and Increasing Local CHI3L1 or LCN2 Levels, Respectively

**Figure 5:**
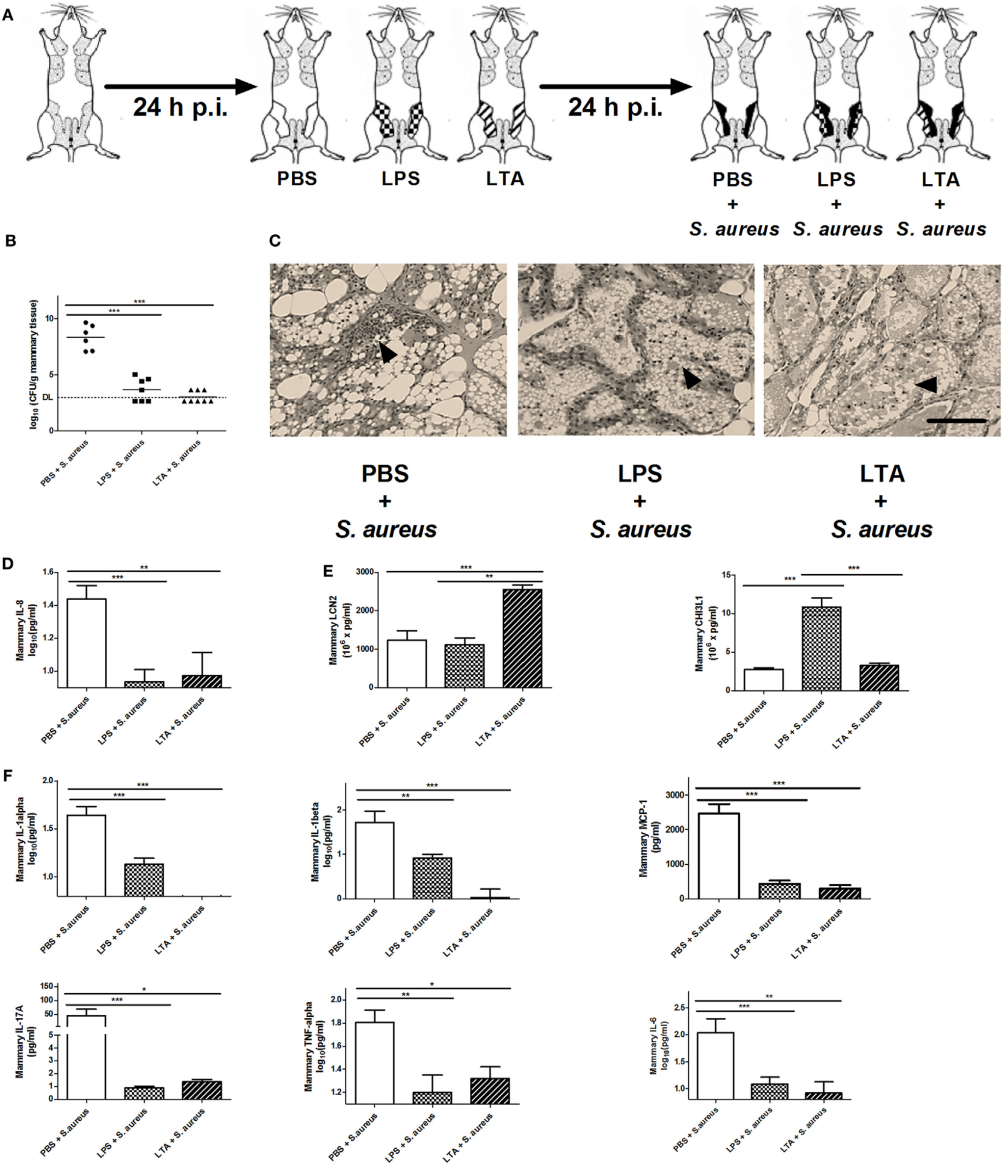

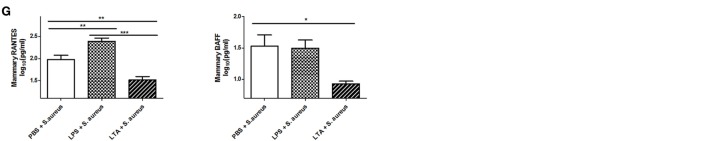
The effect of lipopolysaccharide (LPS) or lipoteichoic acid (LTA) preconditioning on *Staphylococcus aureus* mastitis in mice. **(A)** Schematic presentation of the experimental set-up. Following PBS (white), LPS (checkered pattern) or LTA (striped pattern) preconditioning, mammary glands were inoculated with *S. aureus* (black). **(B)** Bacterial loads in mammary glands that received PBS, LPS, or LTA preconditioning prior to a *S. aureus* infection (PBS + *S. aureus*: *n* = 6 glands; LPS + *S. aureus*: *n* = 7 glands; LTA + *S. aureus*: *n* = 8 glands). **(C)** Representative images of alveoli of mammary glands that had PBS, LPS, or LTA preconditioning prior to a *S. aureus* infection. PMN are indicated by an arrow. Scale bar, 100 µm. **(D)** The IL-8 levels in mammary glands that received PBS, LPS, or LTA preconditioning prior to a *S. aureus* infection (PBS + *S. aureus*: *n* = 14 glands; LPS + *S. aureus*: *n* = 17 glands; LTA + *S. aureus*: *n* = 8 glands). **(E)** Lipocalin 2 (LCN2) and chitinase 3-like 1 (CHI3L1) levels in mammary glands that received PBS, LPS, or LTA preconditioning prior to a *S. aureus* infection (LCN2: *n* = 14 glands for PBS + *S. aureus, n* = 17 glands for LPS + *S. aureus, n* = 8 glands for LTA + *S. aureus*; CHI3L1: *n* = 8 glands for PBS + *S. aureus, n* = 16 glands for LPS + *S. aureus, n* = 17 glands for LTA + *S. aureus*). **(F)** IL-1 alpha, IL-1 beta, MCP-1 IL-17A, TNF-alpha, and IL-6 levels in mammary glands that received clodronate before LPS or LTA preconditioning prior to a *S. aureus* infection (PBS + *S. aureus*: *n* = 14 glands; LPS + *S. aureus*: *n* = 17 glands; LTA + *S. aureus*: *n* = 8 glands). **(G)** RANTES/CCL5, and BAFF levels in mammary glands that received clodronate before LPS or LTA preconditioning prior to a *S. aureus* infection (PBS + *S. aureus*: *n* = 14 glands; LPS + *S. aureus*: *n* = 17 glands; LTA + *S. aureus*: *n* = 8 glands). Represented data were pooled from three independent experiments. p.i., post-inoculation. Values are expressed as mean ± SEM (*P < 0.05, **P < 0.01, and ***P < 0.001).

As shown in Figure 1, mice were intramammary injected with PBS, LPS (10,000 EU), or LTA (10,000 EU) 24 h prior to *S. aureus* infection. With this setup, we wanted to verify whether LPS or LTA preconditioning is able to boost the murine mammary gland microenvironment against a *S. aureus* infection (Figure 5A). All mice pretreated with either LPS or LTA showed a significant decrease in local bacterial loads 24 h p.i. with *S. aureus* compared to PBS (sham)-pretreated mammary glands (Figure 5B). Histology also showed that all conditions were characterized by the presence of PMN in the alveoli of the mammary glands at 24 p.i. with *S. aureus* (Figure 5C). However, on the molecular level, this observation during LPS and LTA pretreatment was accompanied with a significant reduction in mammary IL-8 levels compared to sham preconditioning (Figure 5D).

As previously shown, preconditioning with PBS prior to *S. aureus* infection induced low levels of CHI3L1, but did not induce LCN2 levels in the mammary gland. Preconditioning with 10,000 EU LPS or LTA prior to *S. aureus* infection, on the other hand, boosted either one of these innate immune biomarkers (Figure 5E). More specifically, a significant increase in mammary CHI3L1 levels was detected with LPS preconditioning prior to *S. aureus* infection, whereas a significant increase in mammary LCN2 levels was observed with LTA preconditioning prior to *S. aureus* infection.

Local cytokine profiling showed that the inflammatory response following infection with *S. aureus* was influenced by the LPS and LTA preconditioning at an inoculum dose of 10,000 EU. IL-1 alpha, IL-1 beta, IL-17A, IL-6, MCP-1, and TNF-alpha followed the same pattern as the neutrophil chemoattractant IL-8 profile and were significantly reduced by LPS and LTA preconditioning compared to PBS preconditioning in the *S. aureus*-infected mammary glands (Figure 5F). Mammary RANTES/CCL5 levels were significantly increased by LPS preconditioning compared to the other conditions, while LTA preconditioning significantly reduced local BAFF levels compared to PBS preconditioning (Figure 5G).

### Alveolar Macrophages Have an Immunomodulatory Function during LPS and LTA Preconditioning against *S. aureus*-Induced Mastitis

**Figure 6:**
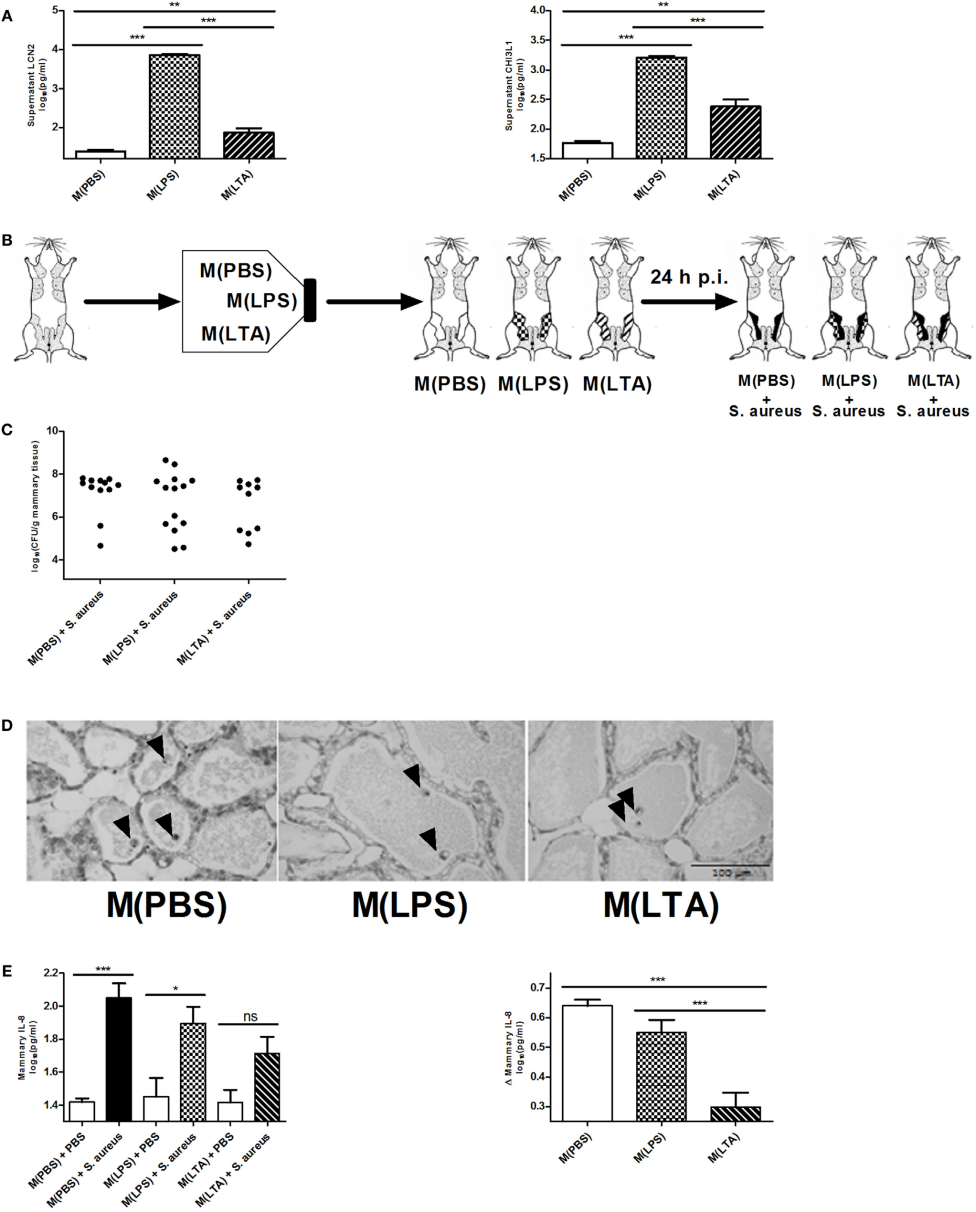

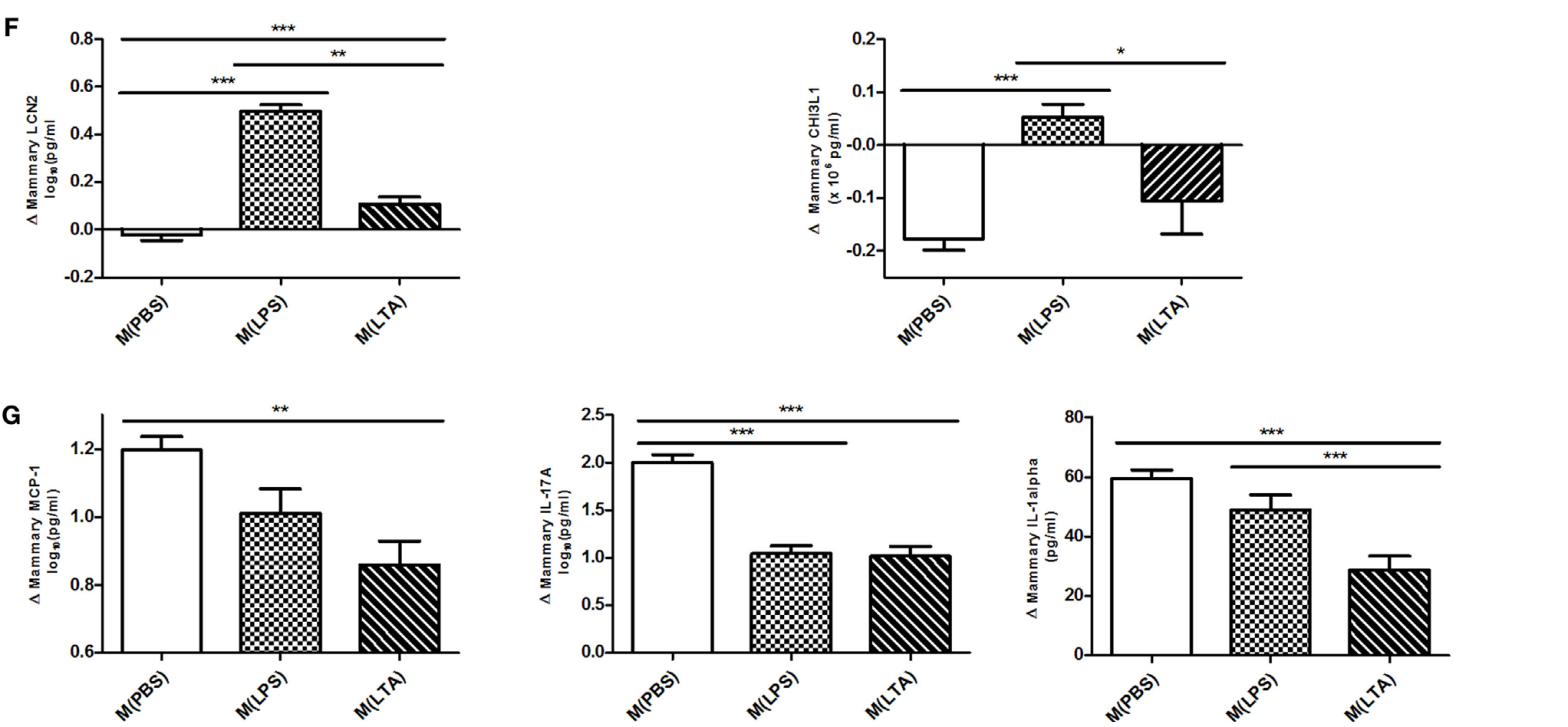
The effect of *ex vivo* activated macrophages on an intramammary infection with *Staphylococcus aureus* in mice. **(A)** Lipocalin 2 (LCN2) and chitinase 3-like 1 (CHI3L1) levels were determined in medium of harvested peritoneal macrophages 24 h post-exposure to PBS [M(PBS)], lipopolysaccharide (LPS) [M(LPS)], or lipoteichoic acid (LTA) [M(LTA)] (*n* = 3 for each group). Represented data were collected from one experiment. **(B)** Schematic presentation of the set-up. Macrophages *ex vivo* exposed to PBS (white glands), LPS or LTA (patterned glands) were transferred into mammary glands and inoculated with PBS (sham) or infected with *S. aureus*. **(C)** Bacterial loads 24 h p.i. with *S. aureus* in mammary glands that received M(PBS), M(LPS), or M(LTA) transfer [M(PBS): *n* = 12 glands; M(LPS): *n* = 14 glands; M(LTA): *n* = 10 glands]. **(D)** Representative images of alveoli of mammary glands that received M(PBS), M(LPS), or M(LTA) transfer followed by PBS (sham). Transferred macrophages are indicated by an arrow. Scale bar, 100 µm. **(E)** Quantitative levels of IL-8 determined in mammary gland tissue that received M(PBS), M(LPS), or M(LTA) transfer followed by an inoculation with PBS (sham) or *S. aureus* (left) [PBS inoculations: *n* = 12 glands for M(PBS), *n* = 13 glands for M(LPS), *n* = 12 glands for M(LTA); *S. aureus* inoculations: *n* = 12 glands for M(PBS), *n* = 6 glands for M(LPS), *n* = 4 glands for M(LTA)]. Normalized quantitative IL-8 values between macrophage transferred glands that received PBS (sham) or *S. aureus*. **(F)** Normalized quantitative LCN2 and CHI3L1 levels between M(PBS), M(LPS), or M(LTA) transferred glands that received PBS (sham) or *S. aureus* [LCN2: *n* = 11 glands for M(PBS), *n* = 14 glands for M(LPS), *n* = 10 glands for M(LTA); CHI3L1: *n* = 12 glands for M(PBS), *n* = 12 glands for M(LPS), *n* = 12 glands for M(LTA)]. **(G)** Normalized quantitative MCP-1, IL-17A, and IL-1 alpha levels between M(PBS), M(LPS), or M(LTA) transferred glands that received PBS (sham) or *S. aureus* [M(PBS): *n* = 17 glands; M(LPS): *n* = 19 glands; M(LTA): *n* = 16 glands]. Represented data were pooled from three independent experiments. p.i., post-inoculation. Values are expressed as mean ± SEM (*P < 0.05, **P < 0.01, and ***P < 0.001).

Alveolar macrophages are a major innate immune cell type in the mammary gland that contain PRRs on the cell surface to sense bacterial antigens (such as LPS or LTA). Macrophages are known to be a crucial cell type during immune tolerance, e.g., endotoxin tolerance. It is a protective mechanism against excessive inflammation where macrophages change their responsiveness to LPS following a first exposure to LPS (50). To test their immunomodulatory potential of macrophages in the mammary gland, isolated murine peritoneal macrophages were exposed *ex vivo* either to 10,000 EU LPS [referred to as M(LPS)], 10,000 EU LTA [referred to as M(LTA)] or PBS [referred to as M(PBS)]. The response of these macrophages was subsequently verified with the two previously used innate immune biomarkers LCN2 and CHI3L1. Activated macrophages, i.e., M(LPS) as well as M(LTA), were found to secrete significantly more LCN2 and CHI3L1 than non-activated macrophages, i.e., M(PBS) (Figure 6A). Of notice, the secreted levels of both these innate immune biomarker proteins showed a significantly higher increase with M(LPS) than with M(LTA).

In a next step, the immune modulating effect of these M(LPS)- versus M(LTA)-activated macrophages was studied in a mammary gland environment. As shown in Figure 1, M(PBS), M(LPS), or M(LTA) were first transferred into murine mammary glands, followed by an inoculation with 10^4^ CFU *S. aureus* bacteria or a PBS (sham) inoculation 24 h later (Figure 6B). The transfer of M(PBS), M(LPS), or M(LTA) did not significantly decrease the bacterial loads in the murine mammary glands at 24 p.i. with *S. aureus*. Nevertheless, there were 40 and 43% of the mammary glands in, respectively, the M(LPS) and the M(LTA) transfer group that had a decreased bacterial load p.i. with *S. aureus* (Figure 6C). In comparison, the number of glands with a decrease in bacterial loads in the M(PBS)-transferred glands was 17%.

The non-significant difference in bacterial loads between the different transfer groups p.i. with *S. aureus* can be reasoned by the lack of PMN recruitment induced by the activated macrophages. Indeed, based on histology, M(PBS), M(LPS), or M(LTA) transfer followed by sham inoculation 24 h later did not induce any recruitment of PMN at 48 h post-macrophage transfer (Figure 6D). The absence of PMN in the alveolar lumen could also be observed based on the low mammary IL-8 levels in the glands that received M(PBS), M(LPS), or M(LTA) followed by sham inoculation (Figure 6E, left). By contrast, the mammary glands that were infected with *S. aureus* at 24 h post-macrophage transfer displayed a significantly increased IL-8 response compared to M(PBS) and PBS (sham). Moreover, statistical analysis showed that the M(PBS), M(LPS), and M(LTA) transfer induced a different IL-8 response in the infected mammary glands. To visualize these differences in IL-8 levels more easily, the effect of macrophage transfer followed by *S. aureus* inoculation in the murine mammary gland was standardized with the IL-8 levels measured following macrophage transfer and subsequent sham inoculation (Figure 6E, right). This analysis showed that immune tolerance was limited with M(LPS) while the transfer of M(LTA) followed by *S. aureus* infection significantly decreased the IL-8 response in the challenged murine mammary glands compared to M(PBS) and M(LPS) transfer and subsequent *S. aureus* infection.

Next, we investigated whether LPS- and LTA-activated macrophages modulate the response of both LCN2 and CHI3L1 *in vivo* to a similar extent as *in vitro*. Standardizing the LCN2 and CHI3L1 levels with M(LPS), M(LTA), and M(PBS) transfer in infected versus non-infected mammary glands showed that M(LPS) transfer significantly boosted both local LCN2 and CHI3L1 levels compared to M(PBS) or M(LTA) transfer upon *S. aureus* infection (Figure 6F). By contrast, but similarly to what was found *in vitro*, M(LTA) induced lower local LCN2 levels following *S. aureus* inoculation, compared to the M(LPS) transfer group. Local CHI3L1 levels, however, were not induced by the M(LTA) transfer group upon *S. aureus* infection (Figure 6F).

Local cytokine profiling further identified a partly and differential immunosuppressive effect due to the activated macrophages (Figure 6G). A significant decrease in the levels of MCP-1, IL-17A, and IL-1 alpha was observed after M(LTA) transfer in the mammary glands p.i. with *S. aureus* compared to M(PBS) transfer. M(LPS) transfer only induced a significant decrease in IL-17A levels compared to M(PBS) transfer. Other cytokines that showed elevated levels at 24 h p.i. with *S. aureus* included IL-6, IL-1 beta, RANTES/CCL5, and BAFF remained unaltered with M(LPS) and M(LTA) transfer compared to M(PBS) transfer (data not shown).

### Macrophage-Independent Signaling Is Important for Bacterial Clearance and Immunomodulation during Mammary Gland Preconditioning with LPS and LTA against *S. aureus*-Induced Mastitis

**Figure 7:**
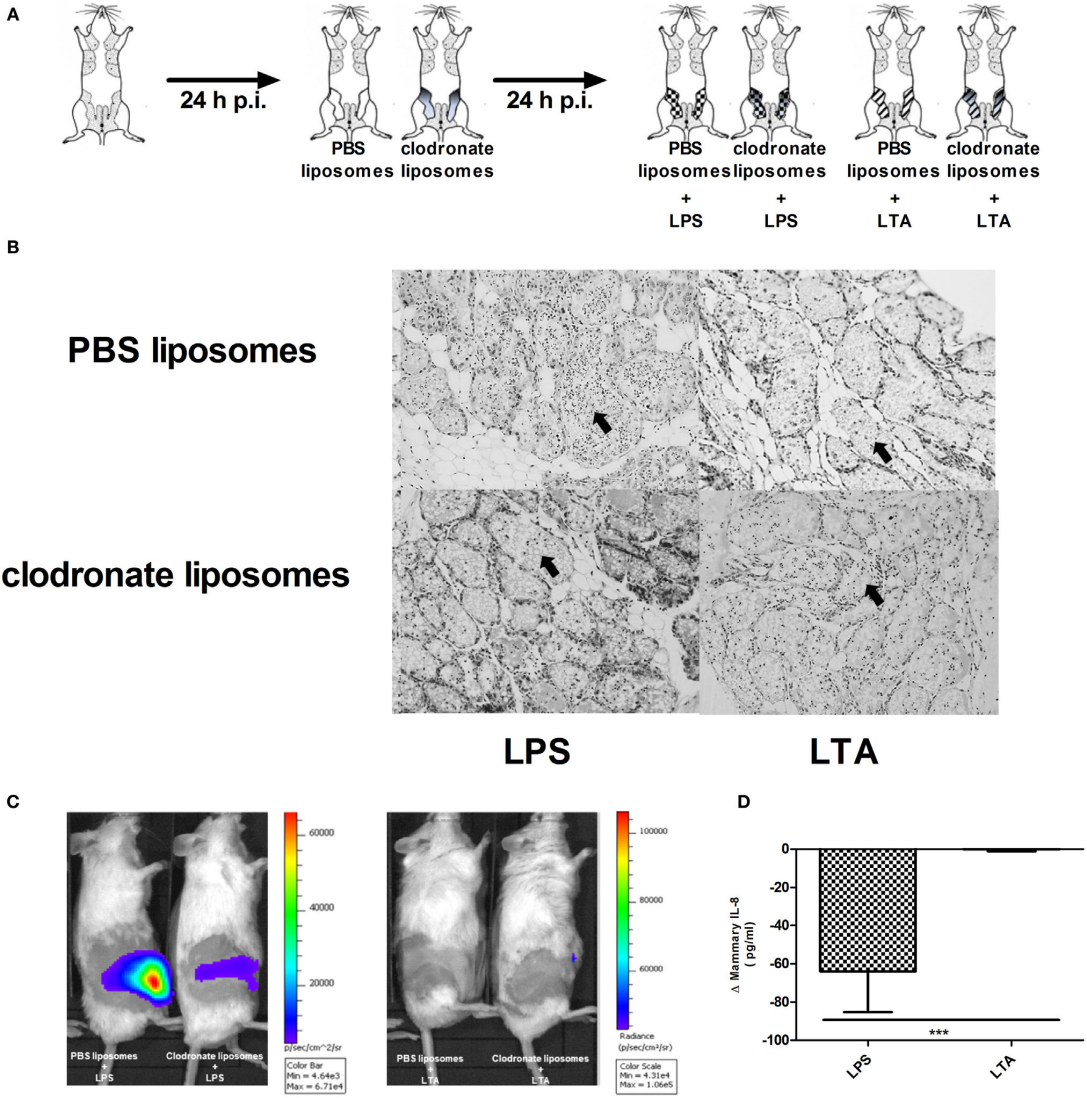
Effect of clodronate liposome treatment on lipopolysaccharide (LPS)- and lipoteichoic acid (LTA)-induced PMN recruitment in the murine mammary gland. **(A)** Schematic presentation of the experimental set-up. The white mammary glands represent the PBS liposome inoculated mammary glands, whereas the gray color gradient glands represent the glands that received clodronate. Following this liposome treatment, mammary glands were inoculated once more with either LPS (checkered pattern) or LTA (striped pattern). **(B)** Representative images of alveoli of mammary glands that received clodronate followed by LPS or LTA. PMN are indicated by an arrow. Scale bar, 100 µm. **(C)** Luminol-based bioluminescence imaging of mouse mammary glands that received clodronate followed by LPS or LTA (*n* = 3 mice for each group). **(D)**. Normalized quantitative IL-8 values between PBS and clodronate inoculation in the mammary glands to compare the differences between LPS or LTA treatment (*n* = 6 glands for each group). Represented data were pooled from three independent experiments. p.i., post-inoculation.

Clodronate liposomes were used to verify the influence of macrophage depletion p.i. LPS or LTA (Figure 7A). Histology showed that intramammary inoculation of clodronate-containing liposomes could decrease the recruitment of PMN in the alveoli of the mammary gland upon challenge with LPS, whereas inoculation of PBS-containing liposomes followed by LPS challenge resulted in a typical strong influx of PMN (Figure 7B). This distinction was not clearly observed when comparing both liposome groups upon challenge with LTA. Luminol-dependent bioluminescence is able to visualize detection of MPO activity and its use *in vivo* is specified for active PMN rather than macrophages. *In vivo* imaging with luminol confirmed the effect of clodronate treatment in the murine mammary gland upon LPS challenge by a decreased MPO activity compared to the mammary glands that were treated with PBS-containing liposomes (Figure 7C). Of note, the low recruitment or activity of PMN by LTA compared to LPS as seen previously, impaired the visualization of the bioluminescent signal from luminol in both conditions. These observations indicate that clodronate treatment has mainly an effect on LPS- rather than LTA-induced inflammation. This was also indirectly shown through the stronger decrease in mammary IL-8 levels following clodronate treatment in the LPS-challenged group compared to the LTA-challenged group (Figure 7D).

**Figure 8:**
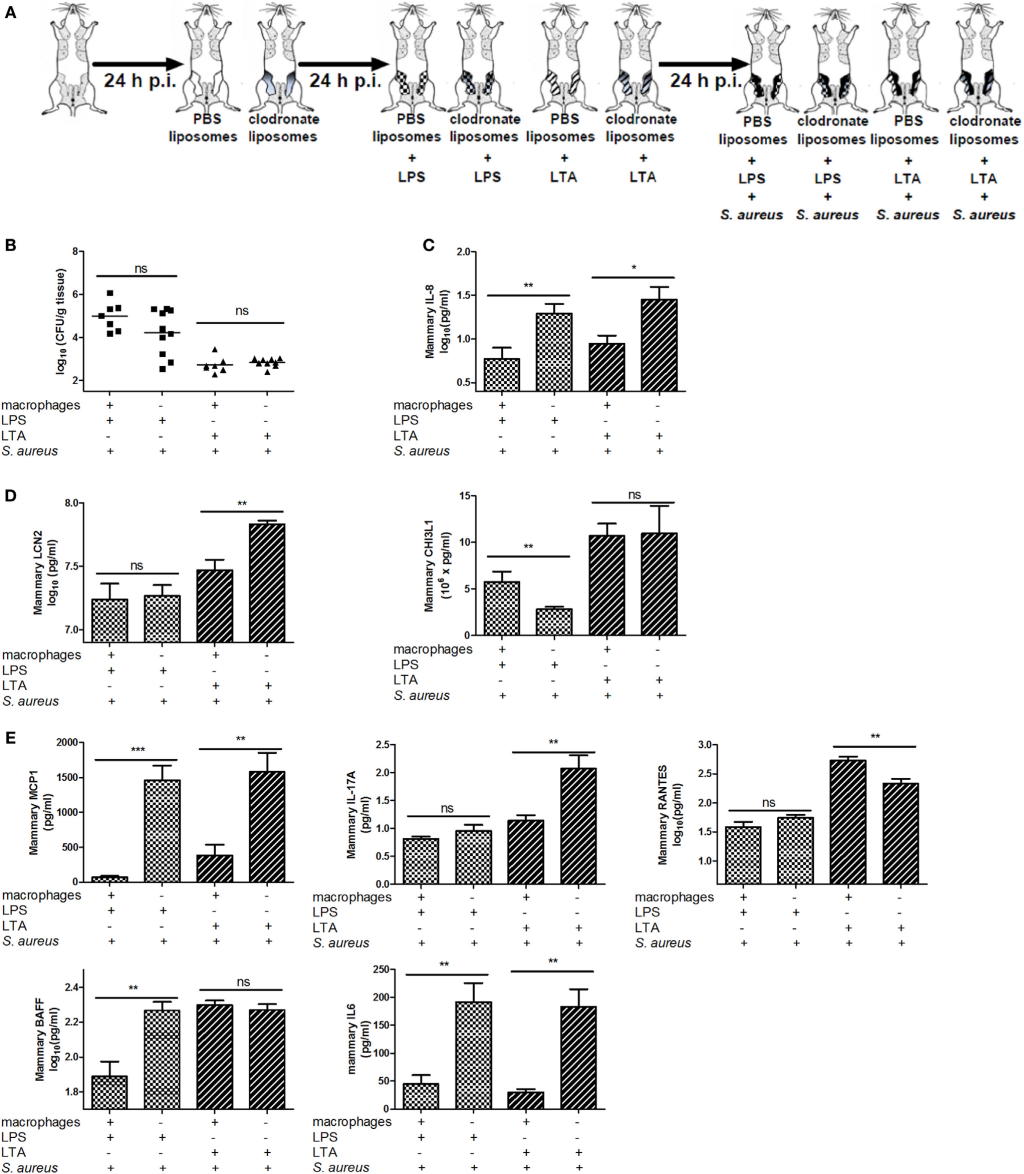
Effect of clodronate liposome treatment on lipopolysaccharide (LPS) and lipoteichoic acid (LTA)—preconditioning to *Staphylococcus aureus* in the murine mammary gland. **(A)** Schematic presentation of the experimental set-up. The treatment groups of Figure 7 received an infection with *S. aureus* (black glands) 24 h p.i. **(B)** The bacterial loads in mammary glands that received clodronate before LPS or LTA preconditioning prior to a *S. aureus* infection (*n* = 7) glands for the PBS liposome groups (i.e., macrophage +); *n* = 10 glands for the clodronate liposome groups (i.e., macrophage −). **(C)** The IL-8 levels in mammary glands that received clodronate injection before LPS or LTA preconditioning prior to a *S. aureus* infection (PBS liposomes: *n* = 8 for the LPS preconditioning group, *n* = 6 for the LTA preconditioning group; clodronate liposomes: *n* = 10 for both the LPS and LTA preconditioning group). **(D)** Lipocalin 2 (LCN2) and chitinase 3-like 1 (CHI3L1) levels in mammary glands that received clodronate injection before LPS or LTA preconditioning prior to a *S. aureus* infection [PBS liposomes: *n* = 7 (LCN2/CHI3L1) for the LPS preconditioning group, *n* = 7 (LCN2) and *n* = 8 (CHI3L1) for the LTA preconditioning group; clodronate liposomes: *n* = 8 (LCN2) and *n* = 10 (CHI3L1) for both the LPS preconditioning group, *n* = 10 (LCN2/CHI3L1) for the LTA preconditioning group]. **(E)** MCP-1, IL-17A, RANTES/CCL5, BAFF, and IL-6 levels in mammary glands that received clodronate injection before LPS or LTA preconditioning prior to a *S. aureus* infection (*n* = 7 glands for the PBS liposome groups; *n* = 10 glands for the clodronate liposome groups). Represented data were collected from one experiment. Values are expressed as mean ± SEM. ns, not statistically significant; p.i., post-inoculation (*P < 0.05, **P < 0.01, and ***P < 0.001).

To verify whether other PRR-expressing mammary cells—next to macrophages—may be activated and have immunomodulatory effects during a *S. aureus* infection, mammary glands of mice were challenged with a clodronate- or PBS-containing liposome treatment followed by either LPS or LTA challenge (both 10,000 EU) and a *S. aureus* infection (Figures 1 and 8A). Local depletion of macrophages by clodronate-containing liposomes did not significantly increase the local bacterial loads when LPS or LTA was intramammarily inoculated prior to *S. aureus* (Figure 8B). However, the innate immune response following *S. aureus* infection was influenced by this depletion as IL-8 levels significantly increased in infected mammary glands challenged with either LPS or LTA (Figure 8C).

In this experimental setup, LCN2 and CHI3L1 also showed different patterns upon *S. aureus* infection in the LPS- versus LTA-challenged groups. Intramammary inoculation with clodronate-containing liposomes and subsequent LTA, but not LPS preconditioning significantly increased local LCN2 levels compared to the PBS liposome treatment in infected glands. By contrast, local CHI3L1 levels were decreased in mammary glands that received LPS preconditioning and clodronate treatment followed by *S. aureus* infection. Liposomes in the mammary gland did not affect the local CHI3L1 levels p.i. with LTA preconditioning followed by *S. aureus* infection (Figure 8D).

Local cytokine profiling also showed that, similar to the neutrophil attractant IL-8 profile, the macrophage chemoattractant MCP-1 as well as IL-6 were increased by the macrophage depletion process both with LPS and LTA preconditioning followed by *S. aureus* infection (Figure 8E). BAFF was only affected by clodronate treatment in the LPS-pretreated group with *S. aureus* infection. IL-17A and RANTES levels, respectively, were significantly increased and decreased, respectively, in the LTA-pretreated group with clodronate-depleted macrophages and *S. aureus* infection.

## Discussion

*Staphylococcus aureus* mastitis is contagious and does not respond well to classical antibiotic therapies (12–14). This Gram-positive pathogen is characterized by biofilm formation and intra-epithelial localization, which hinder access of therapeutic agents (11). Consequently, *S. aureus* udder infections have led to the overuse of antibiotics that increases drug resistance (51–53). Therefore, either novel vaccination or other innovative preventive measures with negligible side-effects are highly anticipated as they have remained unsuccessful in Gram-positive mastitis (7).

The hosts’ recognition of pathogen-associated factors and its innate immune response determines the outcome of bovine mastitis (54, 55). The persistent character of *S. aureus* udder infections is believed to result from a limited mammary cytokine production compared to the “cytokine storm” raised against *E. coli* (20, 21). This implies that *E. coli* antigens are either more easily recognized by mammary PRR or that Gram-negative virulence factors have a higher potency to trigger a strong local immune response. In this study, the dose-dependent stimulation with LPS versus LTA allowed controlled investigation of the potency and differential activation of PRRs through monitoring of local IL-8 production and immune cell infiltration at 24 h p.i. Histology demonstrated that the highest inoculum dose of LPS and LTA initiated innate immune cell recruitment. However, the innate immune response was significantly faster and higher stimulated by LPS compared to LTA.

It has been suggested that LPS may influence the course of a subsequent bacterial udder infection (56, 57). More specifically, injection of LPS in the udder reduced the severity of a subsequent experimental *E. coli* mastitis (57). Whether preconditioning enhances the pathogen recognition or the host immune response is not clear. *Staphylococcus aureus* stimulates TLR2 but lacks efficient activation of TLR4 compared to *E. coli* (58, 59). Similarly, as TLR4 activation by LPS is protective against *E. coli* mastitis, this study aimed to boost TLR2-induced immune response of the murine mammary microenvironment by LTA to protect the host against *S. aureus* mastitis. On the other hand, we also explored the possibility that we should compensate for the lack of TLR4 signaling during *S. aureus* mastitis by preconditioning with LPS. Our results confirmed the observations previously suggested for an *in vitro S. aureus* infection (60, 61): preconditioning the host significantly reduced bacterial loads at 24 h p.i. We also described the participation of local chemo- or cytokines. Although several major local pro-inflammatory mediators decreased, the host was still able to recruit neutrophils to the lumen of LPS or LTA preconditioned alveoli. The lower local IL-8 levels are, therefore, likely an indication of abolished further neutrophil recruitment rather than of their initial LPS- or LTA-induced recruitment. In addition, local MCP-1 and IL-17A levels are signature cytokines for macrophage recruitment and T helper cell activation, indicating that the immunomodulatory response is not limited to neutrophils. On the one hand, LTA but not LPS preconditioning additionally lowered local BAFF levels, a cytokine necessary for proliferation and differentiation of B cells. On the other hand, LPS but not LTA preconditioning elevated local RANTES/CCL5 levels, in line with the *in vitro* observations of cytokine networks after recurrent exposure of bovine MECs to LPS (56). Overall, our different preconditioning protocols against *S. aureus* mastitis together with the reported sterile mammary inflammation data indicate that the two key Gram-negative and -positive virulence factors have unique traits and differentially immunomodulate the mouse mammary gland.

Mammary sentinel cell types—each featuring a distinct immune competence—govern the host response to virulence factors in the mammary gland (16). Some mammary epithelial tissue exposure experiments have described an effect of LPS and LTA on the innate immune response, but how these key virulence factors modulate the mammary gland microenvironment has been scarcely studied *in vitro* and especially *in vivo* (16, 27, 32). The validated murine intramammary inoculation model previously demonstrated that local macrophages are of key importance for the LPS–TLR4 axis-mediated neutrophil recruitment into the alveolar lumen, while this is far less the case for TLR2-mediated signaling (27, 62). In general, macrophages can be programmed to different priming and tolerance states by varying dosages of LPS (63). Partly corroborating the observations from our *in vivo* sterile mammary inflammation data, we demonstrated that macrophages alone affect the mammary environment albeit to a limited degree. Indeed, *in vitro* preconditioning of alveolar macrophages with either LPS or LTA, and their transfer into sterile murine mammary glands did not induce a neutrophil influx. Moreover, upon *S. aureus* infection, activation of these differently preconditioned macrophages reduced the bacterial loads only to a limited extend compared to sham-exposed macrophages. Of relevance, the different TLR trigger changed the response of macrophages as LTA-dependent macrophage signaling was more efficient in reducing the inflammatory signaling compared to the LPS-dependent macrophage signaling.

Injection of macrophages treated with LPS or LTA into the gland prior to infection attenuates the *S. aureus*-induced cytokine release when compared to mice receiving PBS-treated macrophages. However, the pretreated transferred macrophages are not sufficient to reduce the bacterial burden, suggesting that LPS and LTA have to act directly on cells in the mammary gland. MECs have been proposed as special non-professional sentinel cells that are responsible for macrophage-independent local signaling (64). To investigate the role of the mammary epithelium in the LPS- and LTA-mediated immunomodulatory response against *S. aureus*, macrophages were depleted in our preconditioning mastitis model by clodronate-containing liposomes. This local treatment did not change the antibacterial host response, but strongly immunomodulated a subsequent mammary *S. aureus* infection. Consequently, the antibacterial response after preconditioning with either LPS or LTA likely originated from macrophage-independent signaling by the mammary epithelium. Moreover, these data extend those obtained with the preconditioned macrophages. Indeed, macrophages again immunomodulated the mammary compartment during preconditioning as clodronate depletion influenced the local cytokine profile. Depletion of local macrophages was able to further distinguish the differential LPS versus LTA immunomodulatory signaling prior to a *S. aureus* infection. More specifically, our data showed that macrophages inhibited BAFF levels upon LPS preconditioning, while inhibiting IL-17A upon LTA preconditioning. In addition, macrophages had a pro-inflammatory effect by induction of RANTES/CCL5 upon LTA preconditioning prior to *S. aureus* infection. However, based on the methods used in this study, it is not possible to verify whether the changes in cytokine, chemokine, and antimicrobial peptide levels following clodronate pretreatment and subsequent LPS/LTA preconditioning followed by a *S. aureus* infection are either due to an enhanced protein production by the mammary epithelia or the consequence of a reduced uptake by the macrophages after their depletion. Gene expression profiles on isolated mammary cell types would be of added value to distinguish between both phenomena, albeit these were not assessed in this study. In essence, mammary macrophages support the MECs to respond adequately to a local infection. This corroborates the key observations that alveolar macrophages aid the lung epithelia in tissue homeostasis (65) and local immune response (66).

Complementing the local cytokine profiles, we at first evaluated LCN2 and CHI3L1 as potential *in vivo* and *in vitro* innate immunity modulators in the context of mastitis. Both LCN2 and CHI3L1 are stored in granules from mature human neutrophils (67–69). In line with our demonstration of neutrophils on histology, increasing doses of LPS or LTA in the mammary gland comparably induced local LCN2 and CHI3L1 levels. However, no difference was seen at 24 h p.i. for either LPS or LTA at the highest inoculum dose (10,000 EU) as expected based on the differential local IL-8 patterns. Moreover, during *S. aureus*-induced mastitis, a salient finding was that neutrophil recruitment only caused a limited induction of local CHI3L1 and even no increase in mammary LCN2 compared to sham inoculation. These unexpected data strongly indicated that *S. aureus*, in marked contrast to its key virulence factor LTA, did not fully trigger this innate immune signaling even if the basic mastitis characteristic, i.e., neutrophil influx was present. Moreover, our data indicated that neutrophils can be ruled out as the (only) source of LCN2 and CHI3L1, as a strong increase of both innate immune signals was absent in experimental *S. aureus* murine mastitis. Nonetheless, we showed a macrophage-dependent induction of both LCN2 and CHI3L1 in the mammary compartment found to be typically triggered by LPS and less by LTA. Indeed, the latter virulence factor had a remarkably limited influence on the local cytokine profile. By contrast, clodronate treatment demonstrated that the macrophage-independent triggering by LPS was characterized by a strong induction of local CHI3L1 but not of LCN2. Again in marked contrast, LTA now also had a major influence, however, selectively on LCN2 and not on CHI3L1. These important findings strongly indicate that the LPS-induced upregulation of CHI3L1 in the mammary gland is mainly mediated by local macrophages, while the LTA-induced upregulation of LCN2 is inhibited by macrophages. Based on the local cytokine patterns combined with the LCN2 and CHI3L1 levels, a complex interplay between macrophages and the MECs occurs which is of critical importance to effectively induce antibacterial and anti-inflammatory processes and which encompasses either LCN2 or CHI3L1 induction.

In conclusion, extrapolation of our preclinical data allows us to suggest that during a bovine *S. aureus*-induced mastitis, the effective activation of host innate immunity in the murine mammary gland is lacking. We unequivocally demonstrated that activation of the complete mammary gland microenvironment is required to effectively clear this Gram-positive infection and dampen the associated local pro-inflammatory cytokine response. Our current data also underscore the added value of the novel sentinel markers such as LCN2 and CHI3L1 that can be induced by different mammary cell types and may aid in the evaluation of preconditioning protocols.

## Ethics Statement

This study was carried out in accordance with the FELASA guidelines and recommendations. The protocol was approved by the Committee on the Ethics of Animal Experiments of the Ghent University.

## Author Contributions

KB, JS, and KD were essential for the conception and design of the work; the acquisition, analysis, and interpretation of data for the work; and drafted the work. TB and EM revised it critically for important intellectual content. KB, JS, KD, TB, and EM gave their final approval of the version to be published and agreed to be accountable for all aspects of the work in ensuring that questions related to the accuracy or integrity of any part of the work are appropriately investigated and resolved.

## Conflict of Interest Statement

The authors declare that the research was conducted in the absence of any commercial or financial relationships that could be construed as a potential conflict of interest.

## References

[1] Czaplewski L Bax R Clokie M Dawson M Fairhead H Fischetti VA Alternatives to antibiotics – a pipeline portfolio review. Lancet Infect Dis (2016) 16(2):239–51. 10.1016/S1473-3099(15)00466-1 26795692

[2] Maisonneuve E Gerdes K. Molecular mechanisms underlying bacterial persisters. Cell (2014) 157(3):539–48. 10.1016/j.cell.2014.02.050 24766804

[3] Kurtz J. Specific memory within innate immune systems. Trends Immunol (2005) 26(4):186–92. 10.1016/j.it.2005.02.001 15797508

[4] Netea MG Joosten LA Latz E Mills KH Natoli G Stunnenberg HG Trained immunity: a program of innate immune memory in health and disease. Science (2016) 352(6284):aaf1098. 10.1126/science.aaf1098.352/6284/aaf1098 27102489 PMC5087274

[5] Piepers S De Meulemeester L de Kruif A Opsomer G Barkema HW De Vliegher S. Prevalence and distribution of mastitis pathogens in subclinically infected dairy cows in Flanders, Belgium. J Dairy Res (2007) 74(4):478–83. 10.1017/S0022029907002841 17931457

[6] Waage S Mork T Roros A Aasland D Hunshamar A Odegaard SA. Bacteria associated with clinical mastitis in dairy heifers. J Dairy Sci (1999) 82(4):712–9. 10.3168/jds.S0022-0302(99)75288-4 10212457

[7] Barkema HW Schukken YH Zadoks RN. Invited review: the role of cow, pathogen, and treatment regimen in the therapeutic success of bovine *Staphylococcus aureus* mastitis. J Dairy Sci (2006) 89(6):1877–95. 10.3168/jds.S0022-0302(06)72256-1 16702252

[8] Deplanche M Alekseeva L Semenovskaya K Fu CL Dessauge F Finot L *Staphylococcus aureus* phenol-soluble modulins impair interleukin expression in bovine mammary epithelial cells. Infect Immun (2016) 84(6):1682–92. 10.1128/IAI.01330-15 27001539 PMC4907149

[9] Diaz-Murillo V Medina-Estrada I Lopez-Meza JE Ochoa-Zarzosa A Defensin gamma-thionin from *Capsicum chinense* has immunomodulatory effects on bovine mammary epithelial cells during *Staphylococcus aureus* internalization. Peptides (2016) 78:109–18. 10.1016/j.peptides.2016.02.008 26939717

[10] Bardiau M Detilleux J Farnir F Mainil JG Ote I Associations between properties linked with persistence in a collection of *Staphylococcus aureus* isolates from bovine mastitis. Vet Microbiol (2014) 169(1–2):74–9. 10.1016/j.vetmic.2013.12.010 24444863

[11] Melchior MB van Osch MH Lam TJ Vernooij JC Gaastra W Fink-Gremmels J. Extended biofilm susceptibility assay for *Staphylococcus aureus* bovine mastitis isolates: evidence for association between genetic makeup and biofilm susceptibility. J Dairy Sci (2011) 94(12):5926–37. 10.3168/jds.2011-4243 22118083

[12] Barlow J. Mastitis therapy and antimicrobial susceptibility: a multispecies review with a focus on antibiotic treatment of mastitis in dairy cattle. J Mammary Gland Biol Neoplasia (2011) 16(4):383–407. 10.1007/s10911-011-9235-z 21984469

[13] Demon D Ludwig C Breyne K Guede D Dorner JC Froyman R The intramammary efficacy of first generation cephalosporins against *Staphylococcus aureus* mastitis in mice. Vet Microbiol (2012) 160(1–2):141–50. 10.1016/j.vetmic.2012.05.017 22677480

[14] Vanderhaeghen W Cerpentier T Adriaensen C Vicca J Hermans K Butaye P. Methicillin-resistant *Staphylococcus aureus* (MRSA) ST398 associated with clinical and subclinical mastitis in Belgian cows. Vet Microbiol (2010) 144(1–2):166–71. 10.1016/j.vetmic.2009.12.044 20092969

[15] Leitner G Krifucks O Kiran MD Balaban N. Vaccine development for the prevention of staphylococcal mastitis in dairy cows. Vet Immunol Immunopathol (2011) 142(1–2):25–35. 10.1016/j.vetimm.2011.03.023 21524801

[16] Gunther J Koy M Berthold A Schuberth HJ Seyfert HM. Comparison of the pathogen species-specific immune response in udder derived cell types and their models. Vet Res (2016) 47:22. 10.1186/s13567-016-0307-3 26830914 PMC4736154

[17] Porcherie A Cunha P Trotereau A Roussel P Gilbert FB Rainard P Repertoire of *Escherichia coli* agonists sensed by innate immunity receptors of the bovine udder and mammary epithelial cells. Vet Res (2012) 43:14. 10.1186/1297-9716-43-14 22330199 PMC3305352

[18] Blum JW Dosogne H Hoeben D Vangroenweghe F Hammon HM Bruckmaier RM Tumor necrosis factor-alpha and nitrite/nitrate responses during acute mastitis induced by *Escherichia coli* infection and endotoxin in dairy cows. Domest Anim Endocrinol (2000) 19(4):223–35. 10.1016/S0739-7240(00)00079-5 11118787

[19] Gunther J Koczan D Yang W Nurnberg G Repsilber D Schuberth HJ Assessment of the immune capacity of mammary epithelial cells: comparison with mammary tissue after challenge with *Escherichia coli*. Vet Res (2009) 40(4):31. 10.1051/vetres/2009014 19321125 PMC2695127

[20] Gilbert FB Cunha P Jensen K Glass EJ Foucras G Robert-Granie C Differential response of bovine mammary epithelial cells to *Staphylococcus aureus* or *Escherichia coli* agonists of the innate immune system. Vet Res (2013) 44:40. 10.1186/1297-9716-44-40 23758654 PMC3686618

[21] Johnzon CF Artursson K Soderlund R Guss B Ronnberg E Pejler G. Mastitis pathogens with high virulence in a mouse model produce a distinct cytokine profile *in vivo*. Front Immunol (2016) 7:368. 10.3389/fimmu.2016.00368 27713743 PMC5031784

[22] Taponen S Pyorala S. Coagulase-negative staphylococci as cause of bovine mastitis – not so different from *Staphylococcus aureus*? Vet Microbiol (2009) 134(1–2):29–36. 10.1016/j.vetmic.2008.09.011 18977615

[23] Yang W Zerbe H Petzl W Brunner RM Gunther J Draing C Bovine TLR2 and TLR4 properly transduce signals from *Staphylococcus aureus* and *E. coli*, but *S. aureus* fails to both activate NF-kappaB in mammary epithelial cells and to quickly induce TNFalpha and interleukin-8 (CXCL8) expression in the udder. Mol Immunol (2008) 45(5):1385–97. 10.1016/j.molimm.2007.09.004 17936907

[24] Notebaert S Meyer E Mouse models to study the pathogenesis and control of bovine mastitis. A review. Vet Q (2006) 28:2–13. 10.1080/01652176.2006.9695201 16605156

[25] Breyne K Meyer E Infection of the lactating mammary gland: current status on the molecular biology of mastitis. In: Mammary Glands: Anatomy, Development and Diseases (2014). p. 141–72.

[26] Goldammer T Zerbe H Molenaar A Schuberth HJ Brunner RM Kata SR Mastitis increases mammary mRNA abundance of beta-defensin 5, toll-like-receptor 2 (TLR2), and TLR4 but not TLR9 in cattle. Clin Diagn Lab Immunol (2004) 11(1):174–85. 10.1128/CDLI.11.1.174-185.2004 14715566 PMC321333

[27] Elazar S Gonen E Livneh-Kol A Rosenshine I Shpigel NY Neutrophil recruitment in endotoxin-induced murine mastitis is strictly dependent on mammary alveolar macrophages. Vet Res (2010) 41(1):10 10.1051/Vetres/2009058 19828114 PMC2775169

[28] Bauer I Gunther J Wheeler TT Engelmann S Seyfert HM. Extracellular milieu grossly alters pathogen-specific immune response of mammary epithelial cells. BMC Vet Res (2015) 11:172. 10.1186/s12917-015-0489-3 26219462 PMC4518681

[29] Benjamin AL Green BB Hayden LR Barlow JW Kerr DE. Cow-to-cow variation in fibroblast response to a toll-like receptor 2/6 agonist and its relation to mastitis caused by intramammary challenge with *Staphylococcus aureus*. J Dairy Sci (2015) 98(3):1836–50. 10.3168/jds.2014-9075 25597966

[30] Gunther J Czabanska A Bauer I Leigh JA Holst O Seyfert HM *Streptococcus uberis* strains isolated from the bovine mammary gland evade immune recognition by mammary epithelial cells, but not of macrophages. Vet Res (2016) 47:13 10.1186/s13567-015-0287-8 26738804 PMC4704416

[31] Wu JM Ding YL Bi YN Wang Y Zhi Y Wang JL *Staphylococcus aureus* induces TGF-beta(1) and bFGF expression through the activation of AP-1 and NF-kappa B transcription factors in bovine mammary gland fibroblasts. Microb Pathog (2016) 95:7–14. 10.1016/j.micpath.2016.02.013 26948281

[32] Zhang WY Li XZ Xu T Ma MR Zhang Y Gao MQ. Inflammatory responses of stromal fibroblasts to inflammatory epithelial cells are involved in the pathogenesis of bovine mastitis. Exp Cell Res (2016) 349(1):45–52. 10.1016/j.yexcr.2016.09.016 27680776

[33] Ucar A Vafaizadeh V Jarry H Fiedler J Klemmt PA Thum T miR-212 and miR-132 are required for epithelial stromal interactions necessary for mouse mammary gland development. Nat Genet (2010) 42:1101–8. 10.1038/ng.709 21057503

[34] Vidal K Labeta MO Schiffrin EJ Donnet-Hughes A Soluble CD14 in human breast milk and its role in innate immune responses. Acta Odontol Scand (2001) 59:330–4. 10.1080/000163501750541219 11680654

[35] Bachman MA Miller VL Weiser JN Mucosal lipocalin 2 has pro-inflammatory and iron-sequestering effects in response to bacterial enterobactin. PLoS Pathog (2009) 5(10):e1000622 10.1371/journal.ppat.1000622 19834550 PMC2757716

[36] Goetz DH Holmes MA Borregaard N Bluhm ME Raymond KN Strong RK. The neutrophil lipocalin NGAL is a bacteriostatic agent that interferes with siderophore-mediated iron acquisition. Mol Cell (2002) 10(5):1033–43. 10.1016/S1097-2765(02)00708-6 12453412

[37] Sharma N Sodhi SS Kim JH Ghosh M Zhang JJ Koo DB Molecular cloning of lipocalin-2 into a eukaryotic vector and its expression in bovine mammary epithelial cells as a potential treatment for bovine mastitis. Turk J Biol (2016) 40(1):55–68. 10.3906/biy-1501-69

[38] Gao N Yu FSX. Chitinase 3-like 1 promotes *Candida albicans* killing and preserves corneal structure and function by controlling host antifungal responses. Infect Immun (2015) 83(10):4154–64. 10.1128/Iai.00980-15 26238714 PMC4567624

[39] Chiang YC Lin HW Chang CF Chang MC Fu CF Chen TC Overexpression of CHI3L1 is associated with chemoresistance and poor outcome of epithelial ovarian carcinoma. Oncotarget (2015) 6(37):39740–55. 10.18632/oncotarget.5469 26452028 PMC4741859

[40] Dela Cruz CS Liu W He CH Jacoby A Gomitzky A Ma B Chitinase 3-like-1 promotes *Streptococcus pneumoniae* killing and augments host tolerance to lung antibacterial responses. Cell Host Microbe (2012) 12(1):34–46. 10.1016/j.chom.2012.05.017 22817986 PMC3613130

[41] Kamba A Lee IA Mizoguchi E. Potential association between TLR4 and chitinase 3-Like 1 (CHI3L1/YKL-40) signaling on colonic epithelial cells in inflammatory bowel disease and colitis-associated cancer. Curr Mol Med (2013) 13(7):1110–21. 10.2174/1566524011313070006 23170831 PMC3661706

[42] Kawada M Chen CC Arihiro A Nagatani K Watanabe T Mizoguchi E. Chitinase 3-like-1 enhances bacterial adhesion to colonic epithelial cells through the interaction with bacterial chitin-binding protein. Lab Invest (2008) 88(8):883–95. 10.1038/labinvest.2008.47 18490894

[43] Mizoguchi E. Chitinase 3-like-1 exacerbates intestinal inflammation by enhancing bacterial adhesion and invasion in colonic epithelial cells. Gastroenterology (2006) 130(2):398–411. 10.1053/j.gastro.2005.12.007 16472595

[44] Walachowski S Tabouret G Foucras G. Triggering dectin-1-pathway alone is not sufficient to induce cytokine production by murine macrophages. PLoS One (2016) 11(2):e0148464. 10.1371/journal.pone.0148464 26840954 PMC4739705

[45] Mosser DM Edwards JP. Exploring the full spectrum of macrophage activation. Nat Rev Immunol (2008) 8(12):958–69. 10.1038/nri2448 19029990 PMC2724991

[46] Breyne K De Vliegher S De Visscher A Piepers S Meyer E. Technical note: a pilot study using a mouse mastitis model to study differences between bovine associated coagulase-negative staphylococci. J Dairy Sci (2015) 98(2):1090–100. 10.3168/jds.2014-8699 25497801

[47] Peton V Breyne K Rault L Demeyere K Berkova N Meyer E Disruption of the sigS gene attenuates the local innate immune response to *Staphylococcus aureus* in a mouse mastitis model. Vet Microbiol (2016) 186:44–51. 10.1016/j.vetmic.2016.02.014 27016756

[48] Alshetaiwi HS Balivada S Shrestha TB Pyle M Basel MT Bossmann SH Luminol-based bioluminescence imaging of mouse mammary tumors. J Photochem Photobiol B (2013) 127:223–8. 10.1016/j.jphotobiol.2013.08.017 24077442

[49] Breyne K Cool SK Demon D Demeyere K Vandenberghe T Vandenabeele P Non-classical proIL-1beta activation during mammary gland infection is pathogen-dependent but caspase-1 independent. PLoS One (2014) 9(8):e105680. 10.1371/journal.pone.0105680 25162221 PMC4146512

[50] Reschke C Ibrahim D Hecht J Volk H-D Grütz G Epigenetic regulation of cytokine production in sepsis and endotoxin tolerance. Front Immunol (2013). 10.3389/conf.fimmu.2013.02.00573

[51] Chandrasekaran D Venkatesan P Tirumurugaan K Nambi A Thirunavukkarasu P Kumanan K Pattern of antibiotic resistant mastitis in dairy cows. Vet World (2014) 7(6):389–94. 10.14202/vetworld.2014.389-394

[52] Oliver SP Murinda SE Jayarao BM. Impact of antibiotic use in adult dairy cows on antimicrobial resistance of veterinary and human pathogens: a comprehensive review. Foodborne Pathog Dis (2011) 8(3):337–55. 10.1089/fpd.2010.0730 21133795

[53] Willing BF Abundance of Antibiotic Resistance Genes in Feces Following Prophylactic and Therapeutic Intramammary Antibiotic Infusion in Dairy Cattle [Dissertation]. Virginia Tech (2013).

[54] Bannerman DD. Pathogen-dependent induction of cytokines and other soluble inflammatory mediators during intramammary infection of dairy cows. J Anim Sci (2009) 87(13 Suppl):10–25. 10.2527/jas.2008-1187 18708595

[55] Leitner G Krifucks O Glickman A Younis A Saran A. *Staphylococcus aureus* strains isolated from bovine mastitis: virulence, antibody production and protection from challenge in a mouse model. FEMS Immunol Med Microbiol (2003) 35(2):99–106. 10.1016/S0928-8244(02)00458-3 12628544

[56] Gunther J Petzl W Zerbe H Schuberth HJ Koczan D Goetze L Lipopolysaccharide priming enhances expression of effectors of immune defence while decreasing expression of pro-inflammatory cytokines in mammary epithelia cells from cows. BMC Genomics (2012) 13:17. 10.1186/1471-2164-13-17 22235868 PMC3315725

[57] Petzl W Gunther J Pfister T Sauter-Louis C Goetze L von Aulock S Lipopolysaccharide pretreatment of the udder protects against experimental *Escherichia coli* mastitis. Innate Immun (2012) 18(3):467–77. 10.1177/1753425911422407 21990573

[58] Fu Y Zhou E Liu Z Li F Liang D Liu B *Staphylococcus aureus* and *Escherichia coli* elicit different innate immune responses from bovine mammary epithelial cells. Vet Immunol Immunopathol (2013) 155:245–52. 10.1016/j.vetimm.2013.08.003 24018311

[59] Wang H Yu G Yu H Gu M Zhang J Meng X Characterization of TLR2, NOD2, and related cytokines in mammary glands infected by *Staphylococcus aureus* in a rat model. Acta Vet Scand (2015) 20(57):25. 10.1186/s13028-015-0116-0 25990971 PMC4672474

[60] Kauf AC Vinyard BT Bannerman DD. Effect of intramammary infusion of bacterial lipopolysaccharide on experimentally induced *Staphylococcus aureus* intramammary infection. Res Vet Sci (2007) 82(1):39–46. 10.1016/j.rvsc.2006.05.006 16887158

[61] Lohuis JA Kremer W Schukken YH Smit JA Verheijden JH Brand A Growth of *Escherichia coli* in milk from endotoxin-induced mastitic quarters and the course of subsequent experimental *Escherichia coli* mastitis in the cow. J Dairy Sci (1990) 73(6):1508–14. 10.3168/jds.S0022-0302(90)78818-2 2200810

[62] Mintz M Mintz D Ezra-Elia R Shpigel NY. Pam3CSK4/TLR2 signaling elicits neutrophil recruitment and restricts invasion of *Escherichia coli* P4 into mammary gland epithelial cells in a murine mastitis model. Vet Immunol Immunopathol (2013) 152(1–2):168–75. 10.1016/j.vetimm.2012.09.030 23073139

[63] Morris MC Gilliam EA Li L Innate immune programing by endotoxin and its pathological consequences. Front Immunol (2014) 5:680 10.3389/fimmu.2014.00680 25610440 PMC4285116

[64] Atabai K Sheppard D Werb Z. Roles of the innate immune system in mammary gland remodeling during involution. J Mammary Gland Biol Neoplasia (2007) 12(1):37–45. 10.1007/s10911-007-9036-6 17286210 PMC2574498

[65] Han CZ Juncadella IJ Kinchen JM Buckley MW Klibanov AL Dryden K Macrophages redirect phagocytosis by non-professional phagocytes and influence inflammation. Nature (2016) 539(7630):570–4. 10.1038/nature20141 27820945 PMC5799085

[66] Arango Duque G Descoteaux A. Macrophages tell the non-professionals what to do. Dev Cell (2016) 39(6):633–5. 10.1016/j.devcel.2016.12.009 27997821

[67] Borregaard N Cowland JB Granules of the human neutrophilic polymorphonuclear leukocyte. Blood (1997) 89(10):3503–21. 9160655

[68] Nordenbaek C Johansen JS Junker P Borregaard N Sorensen O Price PA. YKL-40, a matrix protein of specific granules in neutrophils, is elevated in serum of patients with community-acquired pneumonia requiring hospitalization. J Infect Dis (1999) 180(5):1722–6. 10.1086/315050 10515841

[69] Volck B Price PA Johansen JS Sorensen O Benfield TL Nielsen HJ YKL-40, a mammalian member of the chitinase family, is a matrix protein of specific granules in human neutrophils. Proc Assoc Am Physicians (1998) 110(4):351–60. 9686683

